# Whole grain intake, diet quality and risk factors of chronic diseases: results from a population-based study in Finnish adults

**DOI:** 10.1007/s00394-023-03272-z

**Published:** 2023-11-07

**Authors:** Rilla Tammi, Satu Männistö, Mirkka Maukonen, Niina E. Kaartinen

**Affiliations:** Department of Public Health and Welfare, Finnish Institute for Health and Welfare (THL), P. O. Box 30, 00271 Helsinki, Finland

**Keywords:** Cardiovascular diseases, Diabetes, Epidemiology, Nordic diet, Nutrition, Obesity

## Abstract

**Objectives:**

Better diet quality of whole grain consumers could contribute to the associations between whole grain intake and chronic disease risk factors. We examined whole grain intake in relation to diet quality and chronic disease risk factors (anthropometrics, blood pressure, cholesterol, triglycerides, C-reactive protein and glucose) and the role of diet quality in whole grains’ associations with each risk factor.

**Methods:**

Our data included 5094 Finnish adults who completed a validated food frequency questionnaire and participated in a health examination within the National FinHealth 2017 Study. We assessed diet quality by the modified Baltic Sea Diet Score. *P* trends were calculated across whole grain intake quintiles by linear regression analysis. Interactions were assessed by including an interaction term in the analyses.

**Results:**

Higher whole grain intake was associated with slightly better diet quality compared with lower intakes in both sexes (*P* < 0.001). Whole grain intake was inversely associated with body mass index (*P* < 0.001), waist circumference (*P* < 0.001) and total cholesterol (*P* = 0.02) in men. Adjusting for medication use attenuated the inverse associations with diastolic blood pressure (*P* = 0.06) and HDL cholesterol (*P* = 0.14) in men. We observed no associations in women. Diet quality did not modify the associations between whole grain intake and chronic disease risk factors.

**Conclusions:**

Our results suggest that whole grain intake was associated with small improvements in the chronic disease risk factors in men, regardless of diet quality. The sex differences may arise from varying health associations of whole grains from different cereal sources.

**Supplementary Information:**

The online version contains supplementary material available at 10.1007/s00394-023-03272-z.

## Introduction

The growing global burden of lifestyle-related chronic diseases calls for changes in food consumption, especially in Western countries. Consistent evidence indicates that increasing whole grain intake benefits weight control and lowers the risk of chronic diseases, such as cardiovascular diseases, type 2 diabetes, and colorectal cancer [1, 2]. Yet, despite recommendations, whole grain intake remains low among most Western populations [3–8].

Whole grain intake may reduce the risk of chronic diseases by pathways related to the regulation of body weight [1], lipid metabolism [9], glucose and insulin responses [10] and chronic low-grade inflammation [11]. So far, evidence of whole grains’ associations with chronic disease risk factors linked to these pathways has been inconsistent in observational [1, 11–16] and interventional studies [2, 9, 11, 17, 18]. Moreover, the components within whole grains that mediate the suggested health effects remain poorly known.

While whole grains may have positive health effects, other factors in diet and lifestyle can also modify whole grains’ health associations. In large observational studies from Europe, Australia and the US, high whole grain intake has been associated with healthier lifestyles, including more physical activity and less smoking [13–15, 19]. Similarly, previous studies have indicated that those with higher whole grain intake tend to consume more of other foods perceived as healthy, such as fruits and vegetables, than those with lower intakes [4, 13, 14, 19]. Thus, the observed health associations could reflect the tendency of whole grain consumers to have an overall healthier lifestyle.

Given the complexity of our diets, examining diet quality along with the intake of individual foods and nutrients could provide a more comprehensive understanding of the relationship between diet and health. In whole grain research, taking diet quality into account could broaden our insight into the dietary factors behind whole grains’ health associations. Thus far, few studies have examined whole grain intake in relation to diet quality assessed by a priori indices. In the US National Health and Nutrition Survey (NHANES) 1999−2004 (*n* = 13 276,  ≥ 19 years), whole grain intake was associated with better diet quality assessed by the Healthy Eating Index [20]. In a study of 20 487 Canadians (≥ 1 year), whole grain *food* consumers had better diet quality, assessed by the Nutrient Rich Food Index, compared with non-consumers [21]. No studies have occurred in Nordic or other European populations. Moreover, although previous studies on whole grain intake and chronic disease risk factors have adjusted analyses for individual foods associated with better diet quality, the role of overall diet quality in the associations has not been examined.

The range of whole grain intake is wide among Finnish adults owing to the central role of whole grain-rich foods, such as rye bread and (oat) porridge, in traditional Finnish diets [22]. Hence, examining whole grain intake in relation to chronic disease risk factors might be more optimal in Finland than in many other Western countries. Consequently, the aim of this cross-sectional study was first to examine whole grain intake in relation to diet quality and chronic disease risk factors (body mass index [BMI], waist circumference [WC], diastolic and systolic blood pressure, total cholesterol, HDL cholesterol, LDL cholesterol, triglycerides [TG], C-reactive protein [CRP], and glucose) in the Finnish adult population. Secondly, we aimed to explore the role of diet quality in the associations of whole grain intake with each chronic disease risk factor.

## Methods

### Study population

We used data from the cross-sectional National FinHealth 2017 Study examining the health, well-being and functional capacity of the Finnish adult population. A nationally representative sample of 10 247 adults aged ≥ 18 years residing in mainland Finland was drawn from the population register by two-staged cluster sampling [23]. Individuals within the sample received an invitation letter to a health examination, including measurements (anthropometrics, blood pressure), blood samples and self-administered questionnaires. Of the invited, 58% participated in the health examination.

Our data comprised individuals who participated in the health examination and completed the food frequency questionnaire (FFQ). Participants received the FFQ in the health examination with oral and written completion instructions. The FFQ was completed either during the examination visit or at home and mailed to the Finnish Institute for Health and Welfare (if on paper). Eighty-six percent of participants who participated in the health examination completed the FFQ. Of those, we excluded participants who withdrew their consent (n = 7), filled the FFQ both on paper and electronically (n = 9), filled the FFQ insufficiently (questionnaire with several blank rows, n = 110) or were pregnant (n = 31). In addition, as a standard procedure to identify participants with extremely implausible food consumption, we excluded 0.5% of the participants in the sex-specific extremes of the daily energy intake distribution range (n = 51). The use of 0.5% is based on previous comparisons between manual identification of implausible food consumption and using a percentage cut-off. Similar methods have been applied elsewhere [24]. After the exclusions, 5094 participants (2844 women and 2250 men) remained in our final data.

This study was conducted according to the guidelines laid down in the Declaration of Helsinki. The Coordinating Ethics Committee at the Hospital District of Helsinki and Uusimaa approved all procedures involving human participants (Reference 37/13/03/00/2016). All participants provided written informed consent.

### Diet

Information on dietary intake over the previous 12 months was gathered by a semi-quantitative 134-item FFQ, which has been repeatedly validated in the Finnish adult population [25–27]. The FFQ inquired about the habitual consumption of commonly used foods, mixed dishes and beverages in Finland [28]. Food consumption was recorded according to ten frequency categories ranging from none to six or more times a day, and each item was allocated a portion size fixed sex-specifically (e.g., glass, slice, volume). To compute the average daily consumption of foods and intake of nutrients (g/d) and energy (kJ/d), an in-house software and the Finnish Food Composition Database Fineli® were used [29].

We assessed whole grain intake using a whole grain database compiled within Fineli® [30]. Whole grains were defined according to the Healthgrain Forum definition as ground, cracked or flaked kernels from which inedible parts are removed and in which bran, endosperm and germ are present in the same relative proportions as in intact kernels [31]. Whole grain cereals included those of the *Poaceae* family, such as wheat, rye, oat, barley and rice, as well as pseudocereals, such as amaranth and buckwheat. Whole grain content was determined for each ingredient, food and mixed dish in the database on a dry matter basis as grams per 100 g of product. We assessed added sugar intake, which was used as a confounder in the analyses, according to a previously published calculation formula [32].

### Diet quality

We assessed diet quality based on the modified Baltic Sea Diet Score (mBSDS), an a priori diet score developed to reflect healthy food choices in the context of a typical Nordic diet [33]. The mBSDS consists of eight score components, including cereals, fruits, vegetables, low-fat milk, fish, red and processed meat, alcohol (ethanol) and fat ratio (ratio of polyunsaturated fatty acids to saturated and *trans*-fatty acids). We excluded cereals from the score to avoid artificially strengthening the association between whole grain intake and the mBSDS. Thus, the score included seven food groups. As in the original Baltic Sea Diet Score, the score was calculated using sex-specific population consumption quartiles by assigning zero to three points for each food group according to the consumption quartile. Higher consumption of fruits, vegetables, low-fat milk and fish, as well as fat ratio, derived higher points, and higher consumption of red and processed meat and alcohol derived lower score points. For alcohol, one point was assigned for consumption within the recommended level in the Nordic Nutrition Recommendations (women ≤ 10 g/d, men ≤ 20 g/d) [34]. As a result, the score could derive a maximum of 19 points, a higher score indicating better alignment with a healthy Nordic diet.

### Sociodemographic and lifestyle factors

Information on participants’ age and sex was derived from the sampling frame. The self-administered questionnaires included questions on education, smoking habits and leisure time physical activity. The participants were categorised into educational tertiles based on self-reported total years of education, considering sex and birth year to adjust for the extension of the basic education system and increase in average school years over time. Smoking status (‘a current smoker’, ‘quit < 6 months ago’, ‘quit ≥ 6 months ago’, ‘a never smoker’) was determined based on answers on smoking history. The categories of ‘quit < 6 months ago’ and ‘quit ≥ 6 months ago’ were combined into one category (‘a former smoker’). Average leisure time physical activity was inquired covering the past 12 months and assessed according to a four-level scale (‘inactive’ [light activities such as reading and watching television], ‘moderately active’ [walking, gardening or other activities ≥ 4 h/week], ‘active’ [running, swimming or other physically demanding activities ≥ 3 h/week], ‘very active’ [competition or other heavy sports several times/week]). The categories of ‘active’ and ‘very active’ were combined into one category (‘active’) due to a small number of ‘very active’ participants.

Information on the current use of lipid-lowering medication (yes or no), antihypertensive medication (never, last time today or yesterday, 2−7 days ago, one week to six months ago, 6−12 months ago, 1−5 years ago,  > 5 years ago) and diabetes medication (no medication, insulin, tablet, insulin and tablet together) was also gathered in the self-administered questionnaires. Regarding antihypertensive medication, we considered medication use within the past seven days to indicate current use. Regarding diabetes medication, we considered the use of either insulin or tablet to indicate current use.

### Anthropometric measures and blood pressure

Trained research staff measured participants’ height (cm), weight (kg), waist circumference (cm) and blood pressure (mmHg) in the health examinations according to standard protocols [35, 36]. Body mass index (BMI) was calculated by dividing weight by height squared (kg/m^2^). A mercury sphygmomanometer was used to measure blood pressure three times from the right arm with participants in a sitting position. The average of the second and third readings was calculated.

### Laboratory analyses

Blood samples were collected at the health examination. The participants were advised to fast and avoid heavy exercise for at least four hours before the examination [23]. Laboratory analyses were conducted in the accredited biochemistry laboratory of the Finnish Institute for Health and Welfare using a clinical chemistry analyser Architect ci8200. Total cholesterol, HDL cholesterol and TG were assessed by the enzymatic Abbott method (Abell-Kendall verification). LDL-cholesterol was calculated using the Friedewald equation [37]. CRP was determined by the immunoturbidimetric Abbott method (ERM-DA472/IFCC). Glucose was measured by enzymatic hexokinase Abbott (NIST SRM 956).

### Statistical analyses

Statistical analyses were performed by IBM SPSS Statistics (version 28; IBM Corp.). We conducted all analyses separately for women and men because whole grain intake differed statistically significantly between the sexes. Means and standard deviations (continuous variables) or percentages (categorical variables) were reported as descriptive statistics of participant characteristics and nutrient intakes. Whole grain intake was adjusted for total energy intake by the residual method [38]. Participants were divided into sex-specific whole grain intake quintiles and sociodemographic and lifestyle factors (age, education, smoking and physical activity), dietary factors (energy intake, mBSDS) and chronic disease risk factors (BMI, WC, diastolic and systolic blood pressure, total cholesterol, HDL cholesterol, LDL cholesterol, TG, CRP and glucose) were examined according to them. Associations of whole grain intake with continuous variables (age, dietary variables, chronic disease risk factors) were tested by linear regression analysis, and associations with categorical variables (education, smoking, physical activity) by logistic regression analysis. In the regression analyses, whole grain intake quintile medians were assigned to the participants and applied as independent continuous variables. Statistical significance was determined as a two-sided *P*-value < 0.05.

When sociodemographic factors, lifestyle factors or mBSDS were examined as outcomes, we adjusted the analyses for age, energy intake, education, smoking, leisure time physical activity and body mass index (fully adjusted model). When chronic disease risk factors were examined as outcomes, the fully adjusted model included further adjustments for the mBSDS (all), sodium intake (blood pressure) and added sugar intake (TG, glucose). The fully adjusted model was applied to report the mean values of each outcome across whole grain intake quintiles to match with the reported p-values. We chose the confounding factors based on the literature.

Two sensitivity models were constructed to further account for energy under-reporting and self-reported use of antihypertensive (blood pressure, CRP), lipid-lowering (cholesterol, TG, CRP) or diabetes (glucose) medication. Under-reporting of energy intake (EI) was determined by calculating the ratio between reported energy intake and predicted basal metabolic rate (BMR) [39]. A value lower than 1.14 indicated energy under-reporting (EI:BMR ≤ 1.14) [40]. To account for the possible confounding of fiber intake in whole grains’ associations with the chronic disease risk factors, we conducted additional analyses including fiber intake in the fully adjusted model.

To further assess whether diet quality modified the association between whole grain intake and the chronic disease risk factors, we stratified the analyses with sex-specific mBSDS tertiles and determined interactions between whole grain intake and mBSDS. Interactions were examined by including an interaction term (whole grain intake*mBSDS tertiles) in the analyses.

## Results

The participants were on average 56 years old and had a mean BMI of 27 kg/m^2^ in women and 28 kg/m^2^ in men (Table 1). Thirteen percent of women and 17% of men were current smokers. Approximately one fourth of women and men were physically inactive. The average daily energy intake was 7.9 MJ in women and 9.7 MJ in men. The mean whole grain intake was 56 g/d (7.0 g/MJ) in women and 65 g/d (6.6 g/MJ) in men.

**Table 1:** Participant characteristics and nutrient intakes (means and standard deviations [SD] or percentages)

	Women (n = 2844)		Men (n = 2250)	
	Mean/%	SD	Mean/%	SD
*Characteristics*				
Age (years)	56	16	56	16
Low educational attainment^a^ (%)	32		32	
Current smokers (%)	13		17	
Physically inactive participants^b^ (%)	26		22	
BMI (kg/m^2^)	27	5	28	4
Energy under-reporters^c^ (%)	36		41	
Antihypertensive medication use (%)	27		28	
Lipid-lowering medication use (%)	15		20	
Diabetes medication use (%)	7		11	
*Energy and nutrient intakes*				
Energy (MJ/d)	7.9	2.6	9.7	3.3
Whole grain (g/d)	56	36	65	45
Whole grain (g/MJ)	7.0	3.9	6.6	3.9
Carbohydrate^d^ (E%)	41	6	40	6
Dietary fiber (g/MJ)	2.8	0.8	2.3	0.7
Added sugar (E%)	7.6	3.5	8.3	3.7
Fat (E%)	37	5	36	5
Protein (E%)	19	3	19	3
Sodium (g/d)	2.7	1.0	3.5	1.3

*BMI* body mass index

^a^Participants were categorized into educational tertiles according to self-reported total years of education. Sex and birth cohort were considered to adjust for the extension of the basic education system and increase in average school years over time

^b^Leisure time physical activity

^c^Under-reporting of energy intake (EI) was determined by calculating the ratio between reported energy intake (EI) and predicted basal metabolic rate (BMR) and applying 1.14 as a cut-off (EI:BMR ≤ 1.14) (39,40)

^d^Available carbohydrate excluding fiber

All associations are reported in the fully adjusted model unless otherwise stated. Higher whole grain intake was associated with older age in both sexes (*P* < 0.001) and lower education in men, compared with lower intakes (*P* = 0.02; Table 2). There were fewer smokers (*P* < 0.01 in women and men) and physically inactive participants (*P* < 0.001 in women, *P* = 0.002 in men) among those with higher whole grain intake compared with those with lower intakes both in women and men. The results remained the same after adjusting for energy under-reporting.

**Table 2:** Sociodemographic and lifestyle factors according to quintiles of whole grain intake (means and standard errors [SE] or percentages)

	Whole grain intake quintiles^a^							
	Q1		Q3		Q5		*P*^c,d^	*P*^c,e^
	Mean^b^/%	SE^b^	Mean/%	SE	Mean/%	SE		
*Age (years)*								
Women	46	0.8	53	0.8	59	0.8	< 0.001	< 0.001
Men	47	0.8	55	0.9	61	0.9	< 0.001	< 0.001
*Low educational attainment*^f^ (%)								
Women	36		28		31		0.69	0.71
Men	36		30		36		0.02	0.01
*Current smokers* (%)								
Women	20		10		9		0.001	0.001
Men	23		15		11		0.004	0.004
*Physically inactive participants*^*g*^ (%)								
Women	32		22		26		< 0.001	< 0.001
Men	30		22		21		0.002	0.002

^a^Quintile medians in women: Q1, 25 g/d; Q2, 51 g/d; Q3, 72 g/d; Q4, 96 g/d; Q5, 132 g/d; in men: Q1, 21 g/d; Q2, 44 g/d; Q3, 65 g/d; Q4, 86 g/d; Q5, 122 g/d

^b^All means and SEs were adjusted for age (years), energy intake (kJ/d), education (tertiles by sex and birth cohort), smoking habits (never, former, current smoker), leisure time physical activity (inactive, moderately active, active) and body mass index (kg/m^2^). A confounder was excluded if it was examined as an outcome

^c^*P* values for trend were tested with linear regression analysis using whole grain intake quintile medians as continuous independent variables

^d^Adjusted for age (years), energy intake (kJ/d), education (tertiles by sex and birth cohort), smoking (never, former or current smoker), leisure time physical activity (inactive, moderately active, active) and body mass index (kg/m^2^) (fully adjusted model). A confounder was excluded from the adjustments if it was examined as an outcome

^e^Fully adjusted model^d^ + energy under-reporting (yes or no)

^f^Participants were categorized into educational tertiles according to self-reported total years of education. Sex and birth cohort were considered to adjust for the extension of the basic education system and increase in average school years over time

^g^Leisure time physical activity

Regarding dietary factors, whole grain intake had a direct association with energy intake in women (*P* < 0.001), whereas no association was observed in men (Table 3). In both sexes, participants with higher whole grain intakes scored on average higher mBSDS points than those with lower intakes (*P* < 0.001 in women and men). Yet, the score difference between the highest (Q5) and lowest (Q1) whole grain intake quintile was only approximately one point in women and two points in men. Of the score components, whole grain intake was directly associated with the consumption of fruits (*P* < 0.01 in women and men) and low-fat milk (*P* < 0.01 in women, *P* < 0.001 in men), and fat ratio (*P* < 0.001 in women and men). The association was inverse with the consumption of vegetables (*P* = 0.02 in women, *P* < 0.01 in men), fish, red and processed meat, and alcohol (all *P* < 0.001 in women and men). The results remained the same after adjusting for energy under-reporting.

**Table 3:** Energy intake, diet quality (mBSDS) and the consumption of the mBSDS components according to quintiles of whole grain intake (means and standard errors [SE])

	Whole grain intake quintiles^a^							
	Q1		Q3		Q5		*P*^c,d^	*P*^c,e^
	Mean^b^	SE^b^	Mean	SE	Mean	SE		
*Energy (MJ/d)*								
Women	7.9	0.2	8.1	0.2	7.3	0.2	< 0.001	< 0.001
Men	9.5	0.2	10.5	0.2	9.2	0.2	0.13	0.07
*mBSDS*								
Women	9.2	0.2	9.5	0.2	10.1	0.2	< 0.001	< 0.001
Men	8.9	0.2	9.9	0.2	10.5	0.2	< 0.001	< 0.001
*Score components (g/d)*								
*Fruits and berries*								
Women	79	6	91	6	96	6	0.001	0.001
Men	62	5	75	5	76	5	0.006	0.006
*Vegetables*								
Women	326	12	302	13	297	13	0.02	0.02
Men	263	10	255	10	237	10	0.005	0.005
*Low-fat milk*								
Women	203	16	226	17	242	17	0.007	0.007
Men	230	20	252	19	315	19	< 0.001	< 0.001
*Fish*								
Women	36	2	30	2	26	2	< 0.001	< 0.001
Men	45	2	44	2	37	2	< 0.001	< 0.001
*Red and processed meat*								
Women	92	3	82	3	69	3	< 0.001	< 0.001
Men	162	5	126	5	109	5	< 0.001	< 0.001
*Fat ratio*^*f*^								
Women	0.47	0.01	0.45	0.01	0.51	0.01	< 0.001	< 0.001
Men	0.43	0.01	0.43	0.01	0.48	0.01	< 0.001	< 0.001
*Alcohol (ethanol)*								
Women	5.7	0.4	5.0	0.4	3.4	0.5	< 0.001	< 0.001
Men	15.7	1.0	10.9	1.0	8.1	1.0	< 0.001	< 0.001

*mBSDS* the modified Baltic Sea Diet Score

^a^Quintile medians in women: Q1, 25 g/d; Q2, 51 g/d; Q3, 72 g/d; Q4, 96 g/d; Q5, 132 g/d; in men: Q1, 21 g/d; Q2, 44 g/d; Q3, 65 g/d; Q4, 86 g/d; Q5, 122 g/d

^b^All means and SEs were adjusted for age (years), energy intake (kJ/d), education (tertiles by sex and birth cohort), smoking (never, former, current smoker), leisure time physical activity (inactive, moderately active, active) and body mass index (kg/m^2^) (fully adjusted model). A confounder was excluded if it was examined as an outcome

^c^*P* values for trend were tested with linear regression analysis using whole grain intake quintile medians as continuous independent variables

^d^Adjusted for age (years), energy intake (kJ/d), education (tertiles by sex and birth cohort), smoking (never, former, current smoker), leisure time physical activity (inactive, moderately active, active) and body mass index (kg/m^2^) (fully adjusted model). A confounder was excluded if it was examined as an outcome

^e^Fully adjusted model^d^ + energy under-reporting (yes or no)

^f^The ratio of polyunsaturated fatty acids to saturated and trans-fatty acids

Of the chronic disease risk factors, whole grain intake was inversely associated with BMI and WC in men (*P* < 0.001; Table 4). The difference in the average BMI between men with the highest and lowest whole grain intakes was approximately 1 kg/m^2^. The average WC was approximately 2.5 cm smaller in men with the highest vs. the lowest whole grain intakes. We also observed indications towards inverse associations between whole grain intake with diastolic blood pressure, HDL cholesterol and total cholesterol in men. The association with diastolic blood pressure was statistically significant in the fully adjusted model and when energy under-reporting was considered (*P* = 0.03) but attenuated after adjusting for antihypertensive medication use (*P* = 0.06). Similarly, the association with HDL cholesterol (fully adjusted model *P* = 0.02; energy under-reporting adjusted model *P* = 0.01) attenuated after adjusting for lipid-lowering medication use (*P* = 0.14). On the contrary, the association with total cholesterol was nonsignificant in the fully adjusted model and after adjusting for energy underreporting (*P* = 0.06) but strengthened after adjusting for lipid-lowering medication use (*P* = 0.02). We observed no associations between whole grain intake and systolic blood pressure, LDL cholesterol, TG, CRP or glucose in men or between whole grain intake and any of the risk factors in women. These results remained the same in the sensitivity analyses.

**Table 4:** Chronic disease risk factors according to quintiles of whole grain intake (means and standard errors [SE])

	Whole grain intake quintiles^a^							
	Q1		Q3		Q5		*P*^c,d^	*P*^c,e^
	Mean^b^	SE^b^	Mean	SE	Mean	SE		
*BMI (kg/m*^*2*^*)*								
Women	27.3	0.38	27.0	0.38	26.9	0.39	0.18	0.28
Men	27.6	0.29	27.6	0.28	26.7	0.29	< 0.001	0.003
*Waist circumference (cm)*								
Women	88.7	0.94	88.0	0.95	87.9	0.96	0.32	0.53
Men	98.4	0.80	98.3	0.77	95.8	0.79	< 0.001	< 0.001
*Diastolic blood pressure (mmHg)*								
Women	75.7	0.74	76.0	0.75	76.5	0.76	0.17	0.16
Men	80.5	0.74	79.9	0.72	79.2	0.73	0.03	0.03
*Systolic blood pressure (mmHg)*								
Women	130.2	1.23	130.1	1.24	130.8	1.26	0.57	0.57
Men	135.6	1.12	135.6	1.09	134.3	1.11	0.27	0.26
*Total cholesterol (mmol/L)*								
Women	5.4	0.08	5.3	0.08	5.3	0.08	0.32	0.33
Men	5.1	0.07	5.0	0.07	5.0	0.07	0.06	0.06
*HDL cholesterol (mmol/L)*								
Women	1.6	0.03	1.6	0.03	1.6	0.03	0.16	0.16
Men	1.4	0.02	1.4	0.02	1.3	0.02	0.02	0.01
*LDL cholesterol (mmol/L)*								
Women	3.1	0.07	3.1	0.07	3.1	0.07	0.33	0.34
Men	3.0	0.06	3.0	0.06	2.9	0.06	0.34	0.35
*Triglycerides (mmol/L)*								
Women	1.3	0.05	1.3	0.05	1.3	0.05	0.24	0.24
Men	1.6	0.07	1.5	0.07	1.6	0.07	0.54	0.54
*C-reactive protein (mg/L)*								
Women	3.5	0.34	3.2	0.34	3.1	0.34	0.46	0.46
Men	2.4	0.41	2.5	0.40	2.5	0.40	0.94	0.95
*Glucose (mmol/L)*								
Women	5.7	0.07	5.6	0.07	5.7	0.08	0.94	0.97
Men	6.1	0.09	5.9	0.09	5.9	0.09	0.12	0.12

*BMI* body mass index

^a^Quintile medians in women: Q1, 25 g/d; Q2, 51 g/d; Q3, 72 g/d; Q4, 96 g/d; Q5, 132 g/d; in men: Q1, 21 g/d; Q2, 44 g/d; Q3, 65 g/d; Q4, 86 g/d; Q5, 122 g/d

^b^All means and SEs were adjusted for age (years), energy intake (kJ/d), education (tertiles by sex and birth cohort), smoking (never, former, current smoker), leisure time physical activity (inactive, moderately active, active), BMI (kg/m^2^), mBSDS (points), sodium intake (g/d, diastolic and systolic blood pressure) and added sugar intake (g/d, TG and glucose) (fully adjusted model). A confounder was excluded if it was examined as an outcome

^c^*P* values for trend were tested with linear regression analysis using whole grain intake quintile medians as continuous independent variables

^d^Adjusted for age (years), energy intake (kJ/d), education (tertiles by sex and birth cohort), smoking (never, former, current smoker), leisure time physical activity (inactive, moderately active, active), BMI (kg/m^2^), mBSDS (points), sodium intake (g/d, diastolic and systolic blood pressure) and added sugar intake (g/d, TG and glucose) (fully adjusted model). A confounder was excluded if it was examined as an outcome

^e^Fully adjusted model^d^ + energy under-reporting (yes or no)

In the additional analyses with the further adjustment for fiber intake, the associations between whole grain intake and BMI, WC and diastolic blood pressure in men were no longer statistically significant (*P* = 0.07, *P* = 0.13, and *P* = 0.14, respectively). The adjustment, however, strengthened the associations between whole grain intake and total and HDL cholesterol in each model in men (*P* < 0.05). Results regarding the other chronic disease risk factors and in women remained the same.

We observed no statistically significant interactions between whole grain intake and the mBSDS in whole grains’ associations with the chronic disease risk factors in either sex. In other words, the associations between whole grain intake and the chronic disease risk factors did not differ according to the level of adherence to the mBSDS (Supplementary Table S1).

## Discussion

In this population-based cross-sectional study of 5094 Finnish adults, higher whole grain intake was associated with slightly better diet quality compared with lower intakes. Both women and men with higher whole grain intakes consumed more fruits and low-fat dairy products, had a better fat ratio, and consumed less red and processed meat and alcohol compared with those with lower whole grain intakes. Of chronic disease risk factors, whole grain intake was associated with lower BMI, diastolic blood pressure, total cholesterol and HDL cholesterol and smaller waist circumference in men, while no associations were observed in women. Diet quality did not modify the associations between whole grain intake and chronic disease risk factors.

Our findings of a direct association between whole grain intake and diet quality are in line with the results in the American (n = 13 276,  ≥ 19 years) and Canadian (n = 13 919,  ≥ 19 years) populations derived using the Healthy Eating Index and Nutrient Rich Food Index, respectively [20, 21]. Consistent with our results, previous studies have also linked whole grain intake with higher consumption of fruits [4, 13, 14, 19] and dairy products [19] and lower consumption of red meat [4, 19] and alcohol [4, 13, 14, 19]. On the other hand, we observed a trend towards lower vegetable and fish consumption among participants with higher whole grain intakes, which is discordant with observations in the British (n = 1521,  ≥ 18 years), American (n = 938, 25−75 years), Australian (n = 7665,  ≥ 18 years) and Scandinavian (n = 8702, 30−65 years) study populations [4, 13, 14, 19]. Even though we adjusted the analyses for education, these inverse associations could be related to other socioeconomic factors, such as income; in a recent study using a sub-sample of our study population (n = 1655), higher cereal consumption was associated with lower income, which, in turn, was associated with lower vegetable and fish consumption [41]. The association between cereal consumption and income likely reflects similar trends between whole grain intake and income, as in Finland, a considerable proportion of cereals are consumed as rye bread and porridge, which are also the main food sources for whole grains [22, 28]. Moreover, consumption of whole grain rye and rye bread have also been associated with lower educational attainment and rural place of residence [22, 42]. In the sub-sample of our study population, lower income was, however, also associated with lower fruit consumption, while in our study, fruit consumption was directly associated with whole grain intake. The association between whole grain intake and fruit consumption could be linked to the traditional Finnish way of eating porridge with berries (included in fruits) for breakfast. In a sub-sample of our study population (n = 1655), porridge contributed to approximately 20% of whole grain intake [22].

Regarding the chronic disease risk factors, we observed inverse associations between whole grain intake and BMI and WC in men but not in women. In accordance with our findings, two earlier studies, one conducted in the Scandinavian countries (n = 8702, 30−65 years) and one in the US (the Health Professionals Follow-up Study n = 468, 40−75 years; the Nurses’ Health Study n = 470, 25−42 years), have reported inverse associations between whole grain intake and BMI only in men [13, 19]. As cereal fiber potentially mediates the associations between whole grain intake and obesity measures [43], the sex differences in our study could be explained by the greater contribution of cereals (and presumably whole grains) on fiber intake in men than women. In a sub-sample of our study population (n = 1655), more than half of fiber intake in men originated from cereals, whereas in women, although cereals were the biggest individual source of fiber, the contributions of vegetables and fruits were also considerable [28]. To consider the possible confounding role of fiber intake, we conducted additional analyses adjusting for fiber intake. Following the adjustment, the associations of whole grain intake with BMI and WC were no longer statistically significant, indicating that the associations were mediated by fiber intake. Similar results have been reported in a study of 7665 Australian adults (≥ 18 years) in which adjustment for cereal fiber intake attenuated the associations of whole grain intake with BMI and WC [14].

As with BMI and WC, we observed inverse associations between whole grain intake and total and HDL cholesterol only in men. These associations were modified by the adjustment for lipid-lowering medication use, which strengthened the association with total cholesterol but attenuated the association with HDL cholesterol. Previous observational studies of whole grain intake and cholesterol have reported both nonsignificant [4, 14, 15] and inverse associations [12, 13], and the findings have been similarly inconsistent after adjusting for lipid-lowering medication use [12, 14, 15]. The differences between men and women in our study could arise from different whole grain sources; in a sub-sample of our study population (n = 1655), rye contributed more to whole grain intake in men than women, whereas oat was a more important whole grain source in women than men [22]. In general, approximately 80% of whole grain intake originated from rye and oat, and the main food sources, rye bread and porridge, covered 80% of whole grain intake. Evidence from randomized controlled trials (RCTs) have suggested that particularly whole grain oat could lower total cholesterol concentrations [9, 44]. Considering this and the higher contribution of oats to whole grain intake in women than men, we would expect the association between whole grain intake and total cholesterol to be stronger in women than men. However, in addition to oat, also rye contains cholesterol-lowering components, such as β-glucans (although less than oat) and arabinoxylans [45], and the lacking evidence of the effects of whole grain rye on cholesterol may be due to less research on the topic. Furthermore, similar to our findings, an older RCT of 40 Finnish adults reported that rye bread consumption reduced total cholesterol in men but not women [46]. This suggests that the sex differences would be attributed to other factors than whole grain sources. Nonetheless, it should be noted that RCTs differ from observational studies in various ways, and the results derived from them are not directly comparable.

Lower total cholesterol among men with higher whole grain intake may not reflect positive changes in cholesterol, given the inverse association of whole grain intake with HDL cholesterol and no association with LDL cholesterol or TG. However, the inverse association between whole grain intake and HDL cholesterol is not supported by previous cross-sectional studies [13–15], and, in our study, the association seemed to be confounded by lipid-lowering medication use. Furthermore, the difference in mean HDL cholesterol concentration between the highest vs. lowest whole grain intake quintile was only 0.04 mmol/L, which may be clinically irrelevant. The mean HDL cholesterol concentration also remained in each whole grain intake quintile above 1.0 mmol/l, which is the reference value for men [47]. Interestingly, the adjustment for fiber intake strengthened the inverse associations between whole grain intake and total and HDL cholesterol in men. Although the health effects of whole grains have mostly been attributed to dietary fiber, several bioactive components within whole grains (e.g., phenolic compounds and phytosterols) may also mediate beneficial effects [48]. As similar results regarding cholesterol have not been reported in previous studies, further research is required to confirm our findings.

Previous studies have suggested that the associations of whole grain intake with chronic diseases and their risk factors might not only be due to whole grains’ own attributes but rather the healthier lifestyle that high whole grain intake seems to reflect [11, 49]. In our study, whole grain intake was associated with less smoking and more leisure time physical activity, along with slightly better diet quality, indicating a healthier lifestyle among those with higher whole grain intakes. This is in accordance with several earlier studies [13, 14, 19]. To account for their confounding effects, we adjusted the analyses of chronic disease risk factors for smoking, physical activity and mBSDS, among other factors. To further elucidate the modifying role of diet quality, we examined interactions between whole grain intake and the mBSDS and stratified the analyses with sex-specific mBSDS tertiles. We observed no evidence that diet quality would modify the associations between whole grain intake and chronic disease risk factors. As the role of diet quality in the association between whole grain intake and chronic disease risk factors has not been examined before, more research is required in different study populations and with appropriate dietary indices to consolidate our findings.

This study has several strengths. We used data from a large, population-based sample of Finnish adults, including comprehensive information on participants’ lifestyles and health collected by self-administered questionnaires and in a health examination. Diet was assessed by a repeatedly validated FFQ, which has been shown to adequately measure carbohydrate fractions in our study population [27]. We assessed diet quality by the mBSDS, which is developed to measure a healthy Nordic diet, hence being appropriate for our study population [33]. The score is, however, calculated based on data-specific cut-offs, which may impair direct comparison between studies. To calculate whole grain intake from the whole diet, we used a recently compiled whole grain database, which includes timely information on whole grain content in each food listed in the FFQ [30]. The relatively high whole grain intake and a wide intake range in our study population provide a good premise for examining associations between whole grain intake and health. Lastly, along with other appropriate adjustments for lifestyle and dietary factors, we tested the associations of whole grain intake with chronic disease risk factors by controlling for fiber intake to highlight the additional benefits of whole grain intake independent of fiber.

Certain limitations also apply. We utilized self-reported dietary intake data, which is prone to misreporting. Nevertheless, to diminish the possible bias, we excluded participants with extremely high or low energy intakes and adjusted the analyses for energy under-reporting. Even though we adjusted the analyses for several previously identified confounders, residual confounding may remain, particularly as all dimensions of healthier lifestyles that whole grain intake might be associate with, cannot be considered. Nonetheless, we endeavoured to examine the confounding role of diet quality in whole grains’ associations with chronic disease risk factors more thoroughly than has been done before. Finally, as this study focused on total whole grain intake, we did not examine whole grain intake from different cereal sources individually. As whole grains from different cereal sources may differ in their health associations, examining them individually could elucidate whole grains’ associations with chronic disease risk factors. Moreover, the generalizability of our results may be affected by the fact that Finnish adults mainly consume whole grains as rye [22], while the main whole grain source in other Western countries is predominantly wheat [3, 5–7, 50]. The health associations of different whole grain cereals are a subject of further research.

To conclude, whole grain intake was associated with slightly more favourable values of some chronic disease risk factors in men and with better diet quality in both sexes. The associations between whole grain intake and the chronic disease risk factors were not, however, attributed to diet quality. To consolidate our findings, more research is needed on the confounding role of diet quality in the associations between whole grain intake and chronic disease risk factors in different study populations. Research is also warranted on the associations of different whole grain cereals with chronic disease risk factors.

### Supplementary Information

Below is the link to the electronic supplementary material.

Supplementary file1 (PDF 180 KB)

## Data Availability

The dataset analysed in the current study is available upon request through the Findata permit procedure at https://findata.fi/en/.
