# Supplementary material for: Whole grain intake, diet quality and risk factors of chronic diseases: results from a population-based study in Finnish adults
Source: Eur J Nutr. 2023 Nov 7;63(2):397–408. doi: 10.1007/s00394-023-03272-z (PMC10899358; doi:10.1007/s00394-023-03272-z)
Supplement: Supplementary file 1 — Supplementary file1 (PDF 180 KB) [file 394_2023_3272_MOESM1_ESM.pdf]

SUPPLEMENTARY MATERIAL

Journal: European Journal of Nutrition

Title: Whole grain intake, diet quality and risk factors of chronic diseases: results from a population-based study in Finnish adults

Authors: Rilla Tammi[1], Satu Männistö[1], Mirkka Maukonen[1], Niina E. Kaartinen[1]

Affiliations: [1]Department of Public Health and Welfare, Finnish Institute for Health and Welfare (THL), Helsinki, Finland

Corresponding author: Rilla Tammi (rilla.tammi@thl.fi)

Supplementary table S1. Interactions by diet quality (mBSDS) in associations between whole grain intake and chronic disease risk factors (means and standard errors [SE])

	Whole grain intake quintiles[a]							
	Q1		Q3		Q5			
	Mean[b]	SE[b]	Mean	SE	Mean	SE	*P*[c,d]	$P_{interaction}$[c,e]
Women[f]								
BMI (kg/m^2)								0.67
mBSDS low	28	0.6	27	0.7	27	0.7	0.28	
mBSDS middle	27	0.6	27	0.6	27	0.6	0.98	
mBSDS high	27	0.9	27	0.9	26	0.9	0.12	
Waist circumference (cm)								0.92
mBSDS low	90	1.5	87	1.6	88	1.6	0.23	
mBSDS middle	88	1.6	87	1.5	88	1.6	0.76	
mBSDS high	88	2.3	89	2.2	87	2.2	0.24	
Diastolic blood pressure (mmHg)								0.46
mBSDS low	74	1.2	76	1.2	75	1.3	0.21	
mBSDS middle	76	1.2	76	1.2	77	1.3	0.43	
mBSDS high	76	1.8	74	1.8	75	1.8	0.64	
Systolic blood pressure (mmHg)								0.10
mBSDS low	128	1.9	130	2.0	127	2.1	0.50	
mBSDS middle	131	2.0	129	2.0	132	2.0	0.77	
mBSDS high	130	3.1	129	3.0	131	3.0	0.27	
Total cholesterol (mmol/L)								0.36
mBSDS low	5.3	0.1	5.4	0.1	5.3	0.1	0.79	

mBSDS middle	5.4	0.1	5.2	0.1	5.3	0.1	0.20	
mBSDS high	5.3	0.2	5.0	0.2	5.1	0.2	0.33	
HDL cholesterol (mmol/L)								0.10
mBSDS low	1.6	0.04	1.6	0.04	1.6	0.04	0.93	
mBSDS middle	1.6	0.04	1.6	0.04	1.6	0.04	0.76	
mBSDS high	1.7	0.06	1.6	0.06	1.6	0.06	0.02	
LDL cholesterol (mmol/L)								0.34
mBSDS low	3.1	0.11	3.2	0.12	3.1	0.12	0.57	
mBSDS middle	3.2	0.11	3.0	0.11	3.1	0.11	0.10	
mBSDS high	3.0	0.16	2.8	0.16	2.9	0.16	0.47	
Triglycerides (mmol/L)								0.10
mBSDS low	1.4	0.09	1.4	0.09	1.3	0.10	0.44	
mBSDS middle	1.2	0.08	1.3	0.08	1.3	0.08	0.16	
mBSDS high	1.2	0.11	1.3	0.11	1.3	0.11	0.10	
C-reactive protein (mg/L)								0.98
mBSDS low	4.6	0.6	4.2	0.6	4.2	0.6	0.69	
mBSDS middle	2.9	0.4	2.8	0.4	3.0	0.4	0.58	
mBSDS high	1.8	0.9	1.8	0.9	1.3	0.9	0.18	
Glucose (mmol/L)								0.77
mBSDS low	5.6	0.1	5.5	0.1	5.6	0.1	0.65	
mBSDS middle	5.8	0.1	5.8	0.1	5.7	0.1	0.91	
mBSDS high	5.7	0.2	5.7	0.2	5.7	0.2	0.85	
Men[g]								
BMI (kg/m^2)								0.62
mBSDS low	27	0.6	27	0.5	27	0.5	0.03	
mBSDS middle	27	0.6	27	0.5	27	0.5	0.13	
mBSDS high	28	0.6	28	0.5	27	0.5	0.05	
Waist circumference (cm)								0.74
mBSDS low	99	1.3	98	1.5	96	1.5	0.01	
mBSDS middle	98	1.5	98	1.4	95	1.5	0.03	
mBSDS high	99	1.6	99	1.3	96	1.3	0.08	
Diastolic blood pressure (mmHg)								0.79
mBSDS low	82	1.2	81	1.4	80	1.4	0.26	
mBSDS middle	79	1.4	80	1.2	77	1.4	0.15	
mBSDS high	81	1.5	79	1.3	79	1.2	0.81	
Systolic blood pressure (mmHg)								0.92
mBSDS low	135	1.7	135	2.0	133	2.0	0.58	

mBSDS middle	137	1.9	137	1.7	133	1.9	0.09	
mBSDS high	136	2.6	134	2.1	135	2.1	0.88	
Total cholesterol (mmol/L)								0.94
mBSDS low	5.2	0.11	5.2	0.13	5.2	0.13	0.24	
mBSDS middle	5.0	0.14	5.0	0.12	4.9	0.13	0.22	
mBSDS high	5.1	0.15	4.9	0.12	4.9	0.12	0.17	
HDL cholesterol (mmol/L)								0.33
mBSDS low	1.4	0.03	1.4	0.04	1.4	0.04	0.22	
mBSDS middle	1.3	0.04	1.4	0.04	1.3	0.04	0.45	
mBSDS high	1.4	0.04	1.4	0.03	1.3	0.03	0.01	
LDL cholesterol (mmol/L)								0.46
mBSDS low	3.0	0.10	3.0	0.11	3.0	0.11	0.99	
mBSDS middle	2.9	0.12	3.0	0.11	2.8	0.12	0.29	
mBSDS high	3.1	0.13	3.0	0.11	3.0	0.11	0.22	
Triglycerides (mmol/L)								0.05
mBSDS low	1.9	0.12	1.8	0.14	1.7	0.14	0.14	
mBSDS middle	1.5	0.13	1.4	0.12	1.5	0.13	0.93	
mBSDS high	1.3	0.11	1.4	0.09	1.4	0.09	0.14	
C-reactive protein (mg/L)								0.24
mBSDS low	3.2	0.7	2.2	0.8	3.5	0.8	0.75	
mBSDS middle	1.8	0.7	2.3	0.7	2.4	0.7	0.94	
mBSDS high	1.7	0.7	2.6	0.6	1.5	0.6	0.77	
Glucose (mmol/L)								0.27
mBSDS low	6.2	0.15	5.9	0.17	5.9	0.17	0.03	
mBSDS middle	5.9	0.17	5.9	0.15	6.0	0.17	0.70	
mBSDS high	6.0	0.18	6.0	0.15	5.9	0.15	0.40	

mBSDS, modified Baltic Sea Diet Score; BMI, body mass index

[a]Quintile medians in women: Q1, 25 g/d; Q2, 51 g/d; Q3, 72 g/d; Q4, 96 g/d; Q5, 132 g/d; in men: Q1, 21 g/d; Q2, 44 g/d; Q3, 65 g/d; Q4, 86 g/d; Q5, 122 g/d

[b]All medians and SEs were adjusted for age (years), energy intake (kJ/d), education (tertiles by sex and birth cohort), smoking (never, former, current smoker), physical activity (inactive, moderately active, active), BMI (kg/m^2), mBSDS (tertiles), sodium intake (g/d, diastolic and systolic blood pressure) and added sugar intake (TG and glucose)

[c]*P* value for trend was tested with linear regression analysis using whole grain intake quintile medians as continuous independent variables

[d]Adjusted for age (years), energy intake (kJ/d), education (tertiles by sex and birth cohort), smoking (never, former, current smoker), physical activity (inactive, moderately active, active), BMI (kg/m^2), mBSDS (tertiles), sodium intake (g/d, diastolic and systolic blood pressure) and added sugar intake (TG and glucose) (fully adjusted model)

[e]Fully adjusted model[d] including an interaction term (whole grain intake*mBSDS tertiles) for the interaction between whole grain intake and diet quality.

[f]The mBSDS tertile cut-offs in women for mBSDS low: ≤8 points; mBSDS middle; 9−11 points; mBSDS high: ≥12 points

[g]The mBSDS tertile cut-offs in men for mBSDS low: ≤8 points; mBSDS middle; 9−11 points; mBSDS high: ≥12 points
